# Effect of the Ketogenic Diet on the Prophylaxis and Treatment of Diabetes Mellitus: A Review of the Meta-Analyses and Clinical Trials

**DOI:** 10.3390/nu15030500

**Published:** 2023-01-18

**Authors:** Damian Dyńka, Katarzyna Kowalcze, Filip Ambrozkiewicz, Agnieszka Paziewska

**Affiliations:** 1 Institute of Health Sciences, Faculty of Medical and Health Sciences, Siedlce University of Natural Sciences and Humanities, 08-110 Siedlce, Poland; 2 Laboratory of Translational Cancer Genomics, Biomedical Center, Faculty of Medicine in Pilsen, Charles University, Alej Svobody 1665/76, 32300 Pilsen, Czech Republic; 3 Department of Neuroendocrinology, Centre of Postgraduate Medical Education, 01-813 Warsaw, Poland

**Keywords:** ketogenic diet, ketosis, diabetes mellitus, type 1 diabetes mellitus, type 2 diabetes mellitus, glycaemia, glycated hemoglobin, HbA1c, HOMA-IR, metabolic syndrome, chronic diseases, obesity, lipid profile, lipidaemia, prophylaxis, treatment, nutrition recommendation, nutrition intervention, ketogenic, dietary patterns, carbohydrates, PRISMA, Cochrane

## Abstract

The exponentially growing frequency of diagnosing diabetes mellitus means that a verification of the previous dietetic approach to treating the disease seems justified. The simultaneous growth of interest in the ketogenic diet and the development of knowledge in this field have contributed to the increasingly frequent application of the ketogenic diet in diabetes treatment. This paper also deals with that issue; its aim includes an extensive analysis of the influence of the ketogenic diet on the prophylaxis and treatment of diabetes. The paper has been prepared based on a wide, meticulous analysis of the available literature on the subject. Among other findings, a favorable effect of that nutrition model has been demonstrated on the values of glycated hemoglobin, glucose, insulin, or other metabolic parameters in diabetes patients. The effect of the ketogenic diet on the pharmacotherapy of type 1 and type 2 diabetes has been presented and compared with the standard nutritional management plan recommended for that disease. Further research is needed in this field, especially studies with a long follow-up period. The discussed articles report interesting therapeutic advantages to the ketogenic diet in comparison with standard diets.

## 1. Introduction

Ongoing progress in medical science has contributed to the increasingly frequent research activities conducted into the ketogenic diet. Starting from 1921, when the diet was used for the first time, the scope of studies on the ketogenic nutrition method has been continuously increasing. Its primary application concerned the treatment of epilepsy and was initially developed over a hundred years ago [1]. The significant effects of that diet, observed in the treatment of epilepsy, even in drug-resistant varieties, have received much attention from both the scientific establishment and the public. Currently, it is still widely used in the treatment of epilepsy; not infrequently, its efficacy exceeds that of pharmacotherapy [2,3,4]. The extraordinary therapeutic effects seen in drug-resistant epilepsy gave a motive for the development of research into the effect of the ketogenic diet in many other domains [5,6,7]. One very interesting and forward-looking approach, from the perspective of progress in medicine, concerns the effectiveness of the ketogenic diet in treating diabetes mellitus.

**Figure 1:**
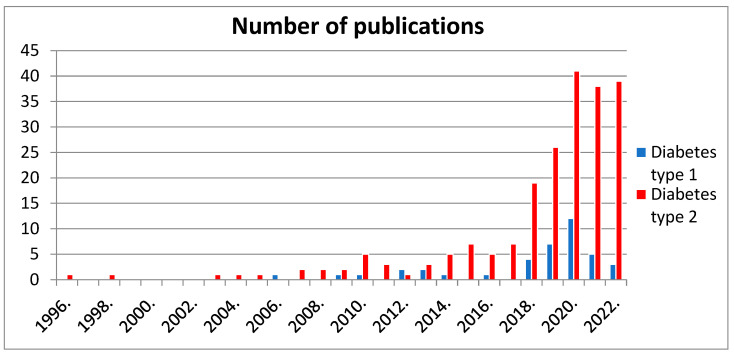
Comparison of the number of publications in the PubMed search engine for type 1 diabetes (phrases: “ketogenic diet and diabetes type 1”; “ketogenic diet and type 1 diabetes”; “ketogenic diet and T1DM”; “ketogenic diet and autoimmune diabetes”) and type 2 diabetes (phrases: “ketogenic diet and diabetes type 2”; “ketogenic diet and type 2 diabetes”; “ketogenic diet and T2DM”).

From the historical point of view, ketogenic diets in T1DM treatment date back to the early twentieth century [8]. The first publication describing the use of a strictly ketogenic diet in type 1 diabetes is the study written by Henwood et al. in 2006 [9]. In terms of T2DM, there is research describing the use of a ketogenic diet as early as 1914–1922 [10]. The diet in question limited the amount of energy from carbohydrates to 8%. Therefore, it can be considered to be a ketogenic diet. The first major study describing the use of a strictly ketogenic diet in type 2 diabetes patients was published in 1996 [11]. Importantly, in both cases, a number of benefits were observed. This encouraged the researchers to further explore the subject, which resulted in a significant increase in the number of studies on this topic in successive years, as shown in Figure 1. The two publications mentioned were the first examples of studies on the effect of a strictly ketogenic diet in type 1 and type 2 diabetes. It is worth emphasizing, however, that recommendations of a diet with the limitation of carbohydrates and an increase in the energy share from fat in diabetes individuals could have also been encountered earlier, in the form of such diets as the paleo, Atkins, South Beach or Zone diets. However, these are not typical ketogenic diets but they show a growing tendency that affects the current studies on strictly ketogenic diets in the treatment of diabetes [12,13,14].

On the one hand, available data may provide indirect evidence in terms of the possible prophylactic application of that diet in diabetes (mainly type 2). Success also depends, to some extent, on its treatment. The results are achieved in the first place from the significant influence of the ketogenic diet on glycemia parameters [15,16,17]. On the other hand, the number of studies suggesting direct benefits in the treatment of diabetes is still insufficient. Other factors, such as the low number of studies on the effect of a ketogenic diet on diabetes, the broad spectrum of available indirect evidence, and the high potential of that diet contribute to the great need for filling the undeveloped scientific space with the development of studies and publications in this area. The multidimensionality and extensiveness of the issue of diabetes, resulting from its various types, also require a clear distinction between studies on the potential use of the ketogenic diet, depending on the type of the disease. Therefore, there is a justified need to conduct research in this area. Taking into account the prevalence of diabetes, such publications could finally lead to the practical implementation of a specific dietetic management plan. This could result in an improvement in the quality of life and the survival of millions of patients struggling with this disease.

## 2. Ketogenic Diet

The ketogenic diet, according to the most accurate and, at the same time, the most general definition, is a diet leading to the increased formation of ketone bodies (such as β-hydroxybutyrate, acetoacetate, and acetone) in the organism. Its task includes somewhat mimicking a condition of fasting, without the negative consequences of starvation [18]. Despite the plethora of various types of diets, this one undoubtedly distinguishes itself from all others. It leads to a change in the body’s preferences concerning its main source of energy supply. While, in other diets, the main source of energy is glucose, in this case, the organism preferentially targets ketone bodies. The organism is then put into the so-called nutritional ketosis state, which, in turn, has many favorable applications [19,20]. It is a diet with a low carbohydrate content, high fat content, and moderate protein content. It can be assumed that the majority of ketogenic diets in practice concern a limitation of the amount of carbohydrates to a maximum of 50 g daily. Moreover, the total supply of carbohydrates can be lowered to 30 g/daily in order to adapt the organism to a more effective use of ketone bodies. However, this is not an elimination diet but only requires reducing to a minimum those products with a higher carbohydrate content. At the same time, the percentage share of fats increases, usually to 70–80%, and protein frequently accounts for about 20% of the energy share. Additionally, the ratio between protein and fat may be more variable and is highly dependent on the specifics of each particular case. This type of theoretical sharing of the macro-components in most cases fits into the frame of the ketogenic diet, but it is worth stressing that, possibly, the least processed products should be used. The products that are most frequently present in such menus include, among other foodstuffs, eggs, meat, and fish (particularly oily fish), plant oils (e.g., olive oil and coconut oil), giblets (e.g., the liver, heart, and kidneys), non-starchy vegetables (all, but primarily the green ones, i.e., broccoli, spinach, lamb’s lettuce, arugula, and kale), avocado, olives, and nuts [21].

## 3. Diabetes Mellitus

Diabetes mellitus is a disease showing a dramatically increasing incidence. The number of diabetes patients worldwide has already exceeded 460 million and is expected to reach 700 million by 2045. This is a very wide field of application, but most frequently, two main diabetes types are distinguished that are characterized by specific mechanisms of development, although the signs are frequently similar [22,23]. Type 1 diabetes mellitus accounts for 5–10% of all cases of the disease and is most frequently diagnosed in children. It is a chronic disease in which the insulin-producing pancreatic beta cells are damaged. Such damage occurs, e.g., as the result of an autoimmune reaction in the body. Because of the absence of a sufficient amount of insulin, the transport of glucose into the cells is impaired and is manifested as elevated serum glucose concentrations [24,25,26]. The manifestations of type 2 diabetes are the same, although the mechanisms of its development are different. In this case, what occurs is an impairment of the response of cells to insulin rather than a shortage of that hormone. The disease usually develops over many years, since it is diagnosed most frequently in adults [27,28]. Criteria for the diagnosis of diabetes mellitus are fasting plasma glucose≥ 126 mg/dL (7.0 mmol/L) or random plasma glucose ≥ 200 mg/dL (11.1 mmol/L) or 2-hour plasma glucose reading with a 75 g oral glucose tolerance test (OGTT) ≥ 200 mg/dL (11.1 mmol/L) or glycated hemoglobin (HbA1c) ≥ 6.5% (48 mmol/mol) [29].

## 4. The Effect of the Ketogenic Diet on the Pharmacotherapy of Type 1 and Type 2 Diabetes

A diagnosis of diabetes is associated with specific, individually selected therapeutic management, including pharmacotherapy. The unusually high prevalence of this subject matter demonstrates that this subject is worth discussing in detail. The results of the available studies suggest an interesting influence of the ketogenic diet on pharmacological treatment. They demonstrate, among other findings, a significant reduction in the body’s requirements for insulin and oral antidiabetic drug doses [30]. It has been shown that the ketogenic diet can reduce the requirement for insulin in type 1 diabetes patients using insulin pumps by as much as 44.3% [31]. It turns out that this value is close to that observed in type 2 diabetes patients, in whom there is frequently a reduction in the requirement for insulin, reducing it by half, on average [32]. The American Association of Clinical Endocrinology suggests, in turn, that patients using a treatment with sodium-glucose cotransporter 2 (SGLT) inhibitors should stop taking them even before the beginning of a ketogenic diet. This is because of the increased risk of the development of diabetes ketoacidosis [33]. Patients receiving treatment with glucagon-like peptide 1 (GLP-1) receptor agonists during ketogenic diet use should be strictly monitored. The necessity for the complete termination of their administration is also suggested. That need is associated, as in the case of SGLT2 inhibitors, with an increased risk not only of diabetes ketoacidosis but also of hypoglycemic episodes [34,35]. In the case of metformin, no general contraindications have been demonstrated, although each case should be individually considered [36]. In that particular nutrition model, it is thus possible to completely withdraw pharmacotherapy or at least to reduce it. A possibility of remission of the disease has also been suggested [37]. That finding was demonstrated in one study, in which most of the patients struggling with type 2 diabetes could have reduced the doses or completely discontinued their treatment with antidiabetic drugs during the 16 weeks of the study’s duration [38]. Taking into account the significant effect of a ketogenic diet on glycemia values and the possibility of reducing drug doses, the authors of the review published in 2021 suggest that the ketogenic diet in type 2 diabetes patients seems to be a promising intervention that can be applied to improve glycemia control [39].

Considering the above-mentioned data, in some patients with pharmacologically treated type 2 diabetes, the prescribed drugs could be completely withdrawn or their doses could be reduced. That would thus contribute to the minimization or avoidance of the potential adverse effects of pharmacotherapy, at least in some patients. Taking into account the high prevalence of the disease, the given proportion of patients would possibly constitute quite a substantial group. It is worth stressing here that the continuous monitoring of the health condition of these patients is extremely important since the subject has not as yet been sufficiently studied. Continuous monitoring and control will enable the modification (or the complete withdrawal) of drug doses in such a way as to prevent episodes of hypoglycemia, diabetes ketoacidosis, and other complications seen in the disease. This is also important from a future perspective when a ketogenic diet could be recommended more frequently in diabetes treatment [30].

## 5. The Effect of the Ketogenic Diet on the Course of Type 1 Diabetes

Studies assessing the effect of a ketogenic diet on diabetes are relatively scarce, and those concerning strictly type 1 diabetes are particularly scarce. On the other hand, diabetes patients and ketogenic diet enthusiasts want to apply it based on certain assumptions, anecdotal evidence, or their own feelings. This is the result of a number of observed potential advantages to a ketogenic diet, which may motivate such individuals to follow the principles of such a diet in spite of a current lack of unequivocal evidence as to its effectiveness in this regard [40]. Therefore, there is a vast gap in the literature for research concerning the effect of a ketogenic diet on diabetes. One of the main causes of the low number of studies in this respect relates to concerns regarding ketoacidosis in diabetes patients who may be persuaded to implement such a type of nutritional management. Frequently, the physiological condition of nutritional ketosis is regarded as a risk factor for ketoacidosis, which can develop, e.g., as the result of type 1 diabetes complications. These two notions should be clearly distinguished. Ketoacidosis is the simultaneous occurrence of very high serum concentrations of ketone bodies (15–25 mmol/L) and glucose (250 mg/dL and higher). That condition leads to a dangerous decrease in blood pH value to a level below 7.3. The physiological condition of nutritional ketosis is characterized by a low value within the normal range (70–99 mg/dL) of glucose concentration and a slight concentration (compared with ketoacidosis) of ketone bodies (usually within the range of 0.5–3 mmol/L). It causes no reduction in blood pH value [19,21,41,42,43]. In the case of healthy individuals, and even in those with type 2 diabetes, the concerns regarding ketoacidosis development due to the adoption of a ketogenic diet are completely irrational and devoid of any scientific foundation. The situation is different in the case of patients suffering from type 1 diabetes. In these patients, such a type of anxiety can be fully justified, and this subject is frequently discussed in the publications concerning that problem. Such publications, among other concerns, also mention the risk of hypoglycemia or dyslipidemia development [44,45].

### 5.1. Possible Mechanisms of Therapeutic Ketogenic Diet Activity in Type 1 Diabetes

There are some circumstances according to which the ketogenic diet can affect type 1 diabetes that is already at the stage of autoimmunization. The autoimmune process, associated with the destruction of pancreatic beta cells, may be related to intestinal homeostasis disorders. These disorders arise, among other factors, from a reduction in the number of intestinal bacteria producing lactate and butyrate, with the simultaneous increase in the number of *Bacteroides* organisms. A protective effect of butyrate is suggested on the autoimmunization process of pancreatic cells. Therefore, it can indirectly prevent the development of type 1 diabetes [46,47]. During ketogenic diet observation, the amount of β-hydroxybutyrate (the main ketone body) increases. On the one hand, it plays a role similar to that of the medium-chain fatty acids produced by intestinal bacteria. On the other hand, it inhibits inflammatory conditions, for example, through the reduction of the number of intestinal proinflammatory Th17 cells [48,49,50]. The extremely widespread effect of the ketogenic diet on the microbiome means that in the future, it would be worth looking at the potential mechanism of its influence on the immunization process, including its effect on pancreatic beta cells [51]. This can also be of importance when taking into account the character of the ketogenic diet itself, which, in principle, should mimic fasting conditions in the body, and which, conversely, is frequently also used in diets employing so-called metabolic windows (e.g., the 16:8 diet). A study in mice demonstrated that a low-carbohydrate, low-protein, and high-fat diet with a calorie deficit was able to affect pancreatic beta cells. It should be stressed, however, that a low-carbohydrate diet is not tantamount to a ketogenic diet. In the case of the pancreatic islets seen in type 1 diabetes, the periods of fasting decrease the activity of PKA and mTOR and induce Sox2 and Ngn3 expression and insulin production. Thus, they promote the reprogramming of the pancreatic islet cells, inducing an expression of genes similar to that observed during fetal life. It has also been demonstrated that they can reverse insulin deficiency in murine and human cells in type 1 diabetes [52,53]. Such a possibility was also suggested by the authors of a study in 2014, in which they presented a case report on a 19-year-old man who had been diagnosed with type 1 diabetes. After the recommendation of a standard diabetes diet on which he consumed 240 g of carbohydrates daily in six meals, over the period of 20 days of diet observation, his glucose concentrations fluctuated within the 68–267 mg/dL range and the general feeling was that he failed to improve. A paleolithic version of the ketogenic diet was then applied. His glucose concentrations had returned to normal already in the first few days; therefore, the administration of insulin was discontinued. After 10 weeks, the testing was repeated to determine C peptide concentration, which increased from the initial 0.6 ng/mL level to 2.2 ng/mL. The patient’s glycated hemoglobin level was 5.5% and his glucose concentration was 88 mg/dL. Although that change could have occurred independently of the diet used, the authors of the study suggest that the evident increase in the C peptide level accompanying insulin withdrawal may indicate the return of insulin production in the pancreas [54]. Many other mechanisms not strictly concerning the autoimmunology process, such as its effect on glucose, insulin, and insulin resistance, are, to a significant degree, common for both types of diabetes. Therefore, the effect on the aspects mentioned is discussed in more detail in Section 6, “Effect of a ketogenic diet on the prevention and treatment of type 2 diabetes mellitus”.

### 5.2. The Ketogenic Diet in the Treatment of Type 1 Diabetes in Children

Based on the conducted studies, the data provided frequently concern individual cases. The available case studies on children with type 1 diabetes who observed a ketogenic diet provide very interesting information. This scenario has been described in, among other papers, the study conducted by McClean et al. [55]. An effective use of the ketogenic diet was demonstrated, among other patients, in a nearly four-year-old boy, suffering from myoclonic-astatic epilepsy and type 1 diabetes, whose glycated hemoglobin values were within the 5.7–6.4% range. No severe hypoglycemia or ketoacidosis episodes were observed. After the institution of the diet, the boy continued to follow it for six years [56]. Another paper presents the case of a 14-year-old girl with type 1 diabetes who observed a direct improvement after beginning a ketogenic diet, an improvement that was also noted in her glucose level measurements [57]. In another study, a girl aged 3.5 years who was suffering from epilepsy and type 1 diabetes was followed up for 15 months while on a ketogenic diet. Since the institution of the diet, no clinical signs of epilepsy had been reported and an improvement in the girl’s development was recorded. Her glycated hemoglobin (HbA1c) level improved, the control of her glycemia was very good, and no severe adverse effects were observed [58]. Another study described the case of a nine-year-old child with type 1 diabetes, in whom, after following a ketogenic diet, insulin administration could have been discontinued [59]. Another two-year-old girl with epilepsy and type 1 diabetes was put on a ketogenic diet. The recorded value of glycated hemoglobin at the time of making the diagnosis was 7.6% and, after six months, it decreased to 6.8%. Over that time period, no severe episodes of ketoacidosis and only many mild hypoglycemia episodes were noted. The girl was put on the diet for 10 months [60]. Interesting data were also provided by a study conducted on a four-year-old girl with pyruvate dehydrogenase deficiency, static encephalopathy, convulsive disorder, and diabetic ketoacidosis. She was simultaneously treated with a ketogenic diet and exogenous insulin, which, at first, seemed quite risky. However, the follow-up period, lasting 28 months, revealed that the ketogenic diet contributed to an improvement in the activity and development of the girl and to the excellent control of glycemia [9]. All the above-mentioned cases are summarized in Table 1.

In 2018 a study was conducted that listed the potential risks resulting from the application of that nutritional model in children with type 1 diabetes, based on six cases. The first of the cases concerned a boy, aged 13 years old, in whom a significant reduction in glycated hemoglobin levels occurred (from 10.3% to 6.3%), while the authors regarded reaching the cholesterol concentration of 5.5 mmol/L as a potentially negative side effect. The second case was that of a 12-year-old girl, in whom the following of a ketogenic diet resulted in a reduction in HbA1c level (from 7.4% to 6%) but hypoglycemia episodes, a cholesterol concentration of 5 mmol/L, and hypertension were observed. The third case described was of a six-year-old boy, in whom a ketogenic diet was associated with hunger, body weight loss, and poor development. The fourth child studied was a girl of four years old, in whom a significant reduction in the HbA1c value (from 14% to 8.1%), many hypoglycemic episodes, slow growth, and delayed bone age were observed. In the fifth case of a 3.5-year-old boy, an HbA1c reduction (from 6.1% to 5.3%), significant hypoglycemia, slower growth, low growth hormone levels, and high cholesterol concentrations were recorded. In the last case concerning a 3.5-year-old girl, unfavorable signs were observed, taking the form of poor body mass gain and disturbed growth [61]. The publication frequently refers to increased total cholesterol levels, which, according to current knowledge, are, rather, of non-significant importance within such a range. The frequent hypoglycemia episodes observed in the study may be an actual problem that is worth keeping in mind; the condition of such patients should be adequately monitored. The authors also refer to the children’s unmet requirements of calcium, in particular, as well as magnesium and phosphorus. These unmet needs, however, are not the effects of the ketogenic diet itself but are rather from an inadequately composed ketogenic diet. Frequently, the diet compensates for the inadequate balance and incorrect nutritional value of the menus that are presented in the studies.

### 5.3. The Ketogenic Diet in the Treatment of Type 1 Diabetes in Adults

The results of the available studies in adults are also promising. In one of these studies on 22 adults, suffering from type 1 diabetes, who decided to go on a ketogenic diet (or, at least, a very low-carbohydrate diet, i.e., 70–90 g of carbohydrates daily), some specific benefits were demonstrated. The joint duration of the follow-up was 12 months; during this period, a significant reduction was observed in hypoglycemic episodes, glycated hemoglobin level (from 7.5% to 6.4%), and the postprandial requirement for insulin by almost half (from 21.1 IU daily to 12.4 IU daily). The total cholesterol and HDL concentration values were not significantly changed, while the concentration of triglycerides alone decreased by 16%, on average [62]. However, it is worth emphasizing, however, that a low-carbohydrate diet was being followed and it was not known whether such an amount was sufficient to induce ketosis, since the level of the ketone bodies was not measured. In the conclusions drawn from another study conducted in 11 adult type 1 diabetes patients who had been on a ketogenic diet for various time periods (2.6 years, on average), the authors demonstrated that a ketogenic diet in patients with type 1 diabetes was, as a rule, associated with normal glycated hemoglobin (HbA1c) concentrations and slight glycemic fluctuations (1.5 ± 0.7 mmol/L). However, they also mentioned that this could have been associated with the large number of hypoglycemia episodes and also with dyslipidemia [63]. Another randomized controlled study that was conducted on adults with type 1 diabetes compared the effect of a low-carbohydrate diet with that of a standard carbohydrate diet over a period of 12 weeks. While, in the carbohydrate group, no changes were observed, in the low-carbohydrate group, a significant reduction in glycated hemoglobin level (from 8.9% to 8.2%), and a reduction in the daily insulin dose (from 64.4 IU to 44.2 IU) and body weight (from 83.2 kg to 78 kg) occurred. Therefore, the authors suggested a certain effectiveness of the diet in type 1 diabetes patients [64]. However, a low-carbohydrate diet is not tantamount to a ketogenic diet, so the results of that study do not necessarily need to be the same as those observed from a ketogenic diet. In 2018, a questionnaire-based survey was distributed to a larger group, i.e., 316 type 1 diabetes patients, with the subjects being on very low-carbohydrate diets (36 g carbohydrate daily on average) for a mean period of 2.2 years. Very low amounts of carbohydrates, on average, are found in the ketogenic diet. It should, however, be kept in mind that these are averaged values. Taking that into account, it is possible that not all study participants entered a state of ketosis. The mean glycated hemoglobin value reported in these patients was 5.67%. Only seven patients gave a history of diabetes-related hospitalizations in the last year: four were due to ketoacidosis, and only two were due to hypoglycemia. The authors concluded that a ketogenic diet affected glycemia control in type 1 diabetes, with a low rate of adverse effects, in both adults and children on a very low-carbohydrate diet (the mean value of 36 g of carbohydrates meets one criterion of a ketogenic diet) [65]. One case study described a 37-year-old male with type 1 diabetes who, in 20 days, had covered a distance of 4011 km on a bicycle in Australia, while being on a ketogenic diet. Continuous glycemia monitoring during the ride revealed an unusual glycemic stability (mean value 6.1 mmol/L) and only one episode of major hypoglycemia. The authors have concluded that this observed glycemic stability suggests that the body’s adaptation to using fats can alleviate glycemia fluctuations. In addition, it can lead to a lower dependence on carbohydrate consumption in order to maintain normal glycemia values during physical exercise [66]. It is worth keeping in mind, however, that an inadequately applied ketogenic diet can be fraught with adverse effects, as was observed in the case of a 22-year-old woman. She was not aware of her type 1 diabetes, and she started to follow a ketogenic diet, which led to diabetic ketoacidosis. That particular case was described in a paper published in 2021 [67]. All the above cases are summarized in Table 2.

## 6. The Effect of the Ketogenic Diet on the Prevention and Treatment of Type 2 Diabetes

In type 2 diabetes, the significantly greater popularity of the ketogenic diet is observed, compared with that in type 1 diabetes. In this case, the occurrence of the possible adverse effects is much less of a concern. Despite that finding, clinicians and scientific societies still cannot find any possible application for it. The favorable mechanisms of a ketogenic diet that have an effect on the prophylaxis and treatment of type 2 diabetes may be multifaceted. Based on the available studies, a conclusion can be drawn that the most important aspect of the issue is the significant effect of this diet on glycemia values.

### 6.1. The Effect of the Ketogenic Diet in the Therapy of Type 2 Diabetes—Meta-Analyses and Systematic Reviews

**Figure 2:**
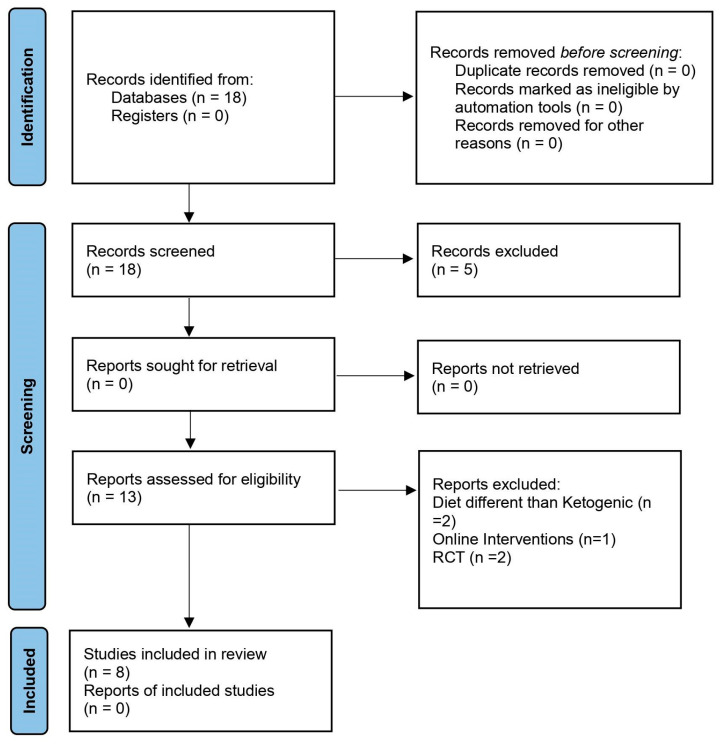
Flowchart presenting the study selection according to PRISMA guidelines.

A number of meta-analyses and systematic reviews on the topic of a ketogenic diet are already available. We considered only those meta-analyses and systematic reviews that were published in English from 2012 to 2022. Search terms included the keywords: “Diabetes” AND Ketogenic Diet”. The literature search was completed on 29 November 2022. This study was performed according to PRISM (preferred reporting items for systemic review) guidelines (Figure 2). The eligibility of studies was assessed by applying clear inclusion/exclusion criteria. The studies included comprised full-text English meta-analyses and systematic reviews on the use of a ketogenic diet by patients with T2DM. Studies excluded were those involving animal studies and those that were focused on other diseases.

A comprehensive meta-analysis of randomized controlled studies demonstrated that the ketogenic diet was more effective than the low-fat diet in terms of the improvement of glycemia parameters, body mass, and lipid profile, particularly in patients with previously diagnosed diabetes who were also overweight [68]. Another meta-analysis concerned the effect of a ketogenic diet on glycemia control, insulin resistance, and lipid metabolism in patients with type 2 diabetes. It was demonstrated that the diet caused an average reduction in glucose concentration by 1.29 mmol/L and a reduction in glycated hemoglobin (% HbA1c) by 1.07% and in the concentrations of total cholesterol, LDL fraction, and triglycerides, as well as an increase in HDL cholesterol concentrations. A reduction in body mass by 8.66 kg, on average, in waist circumference by 9.17 cm, and in BMI by 3.13 kg/m^2^ were also observed. In the conclusion of the study, it was said that the ketogenic diet favorably affected the control of the glycemia and lipid profile in type 2 diabetes patients and, moreover, significantly contributed to body mass reduction [69]. A meta-analysis of randomized controlled trials (RCT) in 2022 also provides important information and presents the benefits of that diet when used for type 2 diabetes. The authors strove for an assessment of its effect on the control of glycemia and body mass in type 2 diabetes patients who were overweight. They found that a ketogenic diet exerted a significant favorable effect on body mass loss (by 5.63 kg, on average), a reduction in waist circumference (by 2.32 cm, on average), a reduction in the concentrations of glycated hemoglobin (by 0.38 HbA1c, on average) and triglycerides (by 0.36 mmol/L, on average) and an increase in HDL cholesterol levels (by 0.28 mmol/L, on average). The authors suggest that the diet can be recommended for type 2 diabetes [70]. Another meta-analysis in 2022 compared the effect of a ketogenic diet with that of a standard diet recommended for diabetes patients. The scope of the study was only limited, however, to an assessment of drug metabolism. The greatest changes were observed in terms of triglyceride concentration reduction in patients on a ketogenic diet. Compared with the control group, an average triglyceride concentration reduction was found in the 3rd, 6th, and 12th months of the treatment, by −0.49 mmol/L, −0.82 mmol/L, and −0.18 mmol/L, respectively. The greatest change was found in the 3rd month. Importantly, however, no significant differences were demonstrated in the total cholesterol, HDL, and LDL concentrations. The authors stressed that type 2 diabetes patients on a ketogenic diet had no elevated total cholesterol and LDL concentrations. Similarly, in the studies included in the meta-analysis, no reduced cholesterol concentrations were observed in the patients [15]. In 2022, a systematic review with a meta-analysis was conducted, in order to estimate the effect of a ketogenic diet in patients with type 2 diabetes and in those with a pre-diabetic condition. The control group in the studies mentioned consisted of individuals following a diet with a higher content of carbohydrates than a ketogenic diet. It was shown that in the patients on a ketogenic diet, compared with the control group, the concentration of triglycerides decreased by −0.28 mmol/L, while that of HDL cholesterol increased by 0.04 mmol/L. In four studies, the changes in glycated hemoglobin concentration after 12 months, with the estimation of permanent effects (very low carbohydrate/ketogenic diets (VLC/KD) minus the control group), were 0.01% (−0.22 to 0.25). In two studies, an HbA1c change of −0.65% (−0.99l–0.31) was noted in relation to the initial value. The authors concluded that a ketogenic diet could effectively reduce HbA1c and triglyceride concentrations in individuals with type 2 diabetes and those in a pre-diabetic condition. They stress, however, that still more well-planned studies are needed [71]. The benefits resulting from a ketogenic diet were also confirmed by another meta-analysis conducted in 2022. The authors compared the effect of the ketogenic diet with that of the standard diets recommended for diabetes patients. Compared with the diets in the control groups, in patients on a ketogenic diet, the following were observed: an HbA1c concentration reduction after three months (by 6.7 mmol/mol on average) and after six months (by 6.3 mmol/mol on average), a body mass reduction after three months (by 2.91 kg, on average) and after six months (by 2.84 kg, on average). Moreover, for 12 months, the diet demonstrated an advantage over the control diets in respect of triglyceride level reduction, HDL cholesterol concentration increase, and a reduction in the administration of antidiabetic drugs. The authors clearly stated, however, that the quality of the currently available evidence was still insufficient to allow for the standard recommendation of a ketogenic diet in patients with type 2 diabetes [72]. In a systematic review with a meta-analysis published in 2022, the advantages of a ketogenic diet were also demonstrated in type 2 diabetes patients. Compared with the control diets, the individuals following a ketogenic diet decreased their glycated hemoglobin values by 1.45% HbA1c on average and reduced their body mass by 2.67 kg on average [73]. This finding was confirmed by another systematic review [39]. It demonstrated that a ketogenic diet improved HbA1c concentration in diabetes patients already showing changes after three weeks; the effect was maintained for at least a year. That change was also associated with a reduction in administered antidiabetic drugs and the sustained persistence of a reduced body mass. In a systematic review of 2022, the effect was studied of a ketogenic diet on insulin sensitivity in type 2 diabetes patients. Once again, its favorable effect on glycemia was confirmed [74]. Another review also suggested its benefits when treating patients with metabolic diseases, such as type 2 diabetes. The authors demonstrated its advantages in respect of reducing the concentrations of HbA1c, glucose, insulin, total cholesterol, triglycerides, ALT, AST, and the HOMA-IR index. It is worth stressing the finding that no severe adverse effects were noted [75]. A summary of the results of all analyses and systematic reviews is shown in Table 3.

### 6.2. The Effect of the Ketogenic Diet in the Therapy of Diabetes Type 2—Randomized Controlled Trials (RCT)

A literature search of the Cochrane Library and PubMed was performed to identify randomized controlled trials. Search terms included the keywords: “Diabetes” AND Ketogenic Diet” in the publications’ titles, abstracts, and keywords. Search filters were set as “Trials” and a time range was set up from 2012 until 2022. The initial database search yielded 41 papers (with 17 papers in PubMed and 25 from the Cochrane Library), from which 17 duplicates were excluded. The remaining 24 trials underwent further screening of the titles and abstracts. As a result, 17 articles were excluded for the following reasons: other co-morbidities (5), no RCT (4), an animal-based study (1), and unrelated topics (7). Full-text examination by two independent reviewers narrowed down the number of articles to 7. The available data from randomized controlled trials (RCT) shows a positive prognosis for the ketogenic diet’s application in diabetes. One such study demonstrated an advantage of a ketogenic diet over a diet composed according to the dietary recommendations of the American Diabetes Association (ADA). It was conducted for 32 weeks in patients with type 2 diabetes who were overweight, as part of an online intervention. In the group on a ketogenic diet, a greater reduction in glycated hemoglobin concentrations (−0.8%, on average) occurred, compared with the other group (−0.3%, on average). Besides that, in 55% of patients, a reduction in the glycated hemoglobin value below 6.5% was found, which was not observed in any subject in the control group. The improvement of triglyceride concentrations was also more pronounced in the group on a ketogenic diet (−60.1 mg/dL, on average) than in the other group (−6.2 mg/dL, on average). Significant differences were also observed in body mass, the mean loss of which was 12.7 kg in the ketogenic diet group and 3 kg in the control group. Importantly, the individuals on a ketogenic diet showed non-compliance significantly less frequently in further adhering to the recommendations (8% of the individuals) compared with those on the control diet (46% of the subjects) [77,78]. Another randomized controlled study also demonstrated an advantage to the low-calorie ketogenic diet over the standard low-calorie diet in 89 adults with type 2 diabetes. A similar level of safety of use was also observed. However, the values of glycated hemoglobin showed greater improvement in the ketogenic diet group. In addition, a significantly greater bodyweight loss and waist circumference reduction were observed [79]. In 2022m a 12-week-long, randomized controlled study was conducted on 60 adult patients with newly diagnosed diabetes who were overweight. The effect of the usual diabetic diet (30 subjects) was compared with that of a ketogenic diet (30 subjects). It was found that improvements in the values of such parameters as glycated hemoglobin (HbA1c), fasting glucose and insulin levels, lipid profile, body mass, and BMI were more pronounced in those individuals on the ketogenic diet. Thus, it demonstrated its higher effectiveness compared with the standard diets [80]. An RCT from 2022 compared the effects of ketogenic and low-carbohydrate Mediterranean diets, along with their influence on glucose concentration and cardio-metabolic risk factors in T2DM or pre-diabetic patients. Both types of diet had a positive effect on HbA1c. However, the ketogenic diet showed greater benefits by decreasing triglyceride concentrations and body weight and increasing HDL cholesterol concentration. In addition, increases of LDL cholesterol was observed, and the authors pointed out a lower balance in the diet [81]. Another study compared the adherence to both diet types in patients with T2DM and pre-diabetes. The results showed a high similarity in diet adherence in the first 4 weeks (when all food was delivered to the participants). Conversely, when the participants were responsible for providing and preparing their own meals, this similarity was lower. In the context of comparing the ketogenic diet to ADA guidelines, data published in 2014 showed the advantages of this nutrition model. It was demonstrated that a higher percentage of participants (44% vs. 11%) could discontinue one or more oral diabetes medications. Additionally, HbA1c and body weight decreased on a ketogenic diet. [82]. Moreover, in a 12-month timeframe, participants on a ketogenic diet showed greater reductions than participants on a low-fat diet. Furthermore, greater reductions in glycated hemoglobin j (−0.5% vs. −0.2%) and weight (−7.9 kg vs. −1.7 kg) were observed. Reductions in diabetes-related medications were only observed for the ketogenic diet [83]. Details of the described RCT are shown in Table 4.

The most promising results were obtained in the RCT conducted by Myette-Côté et al., although considering the fact that this trial did not focus on the ketogenic diet (but on restricting energy from carbohydrates to 10%), it was excluded from Table 4. The aforementioned study compared the short-term influence of three different types of intervention: a low-fat low-glycemic index diet (GL), a low-carbohydrate high-fat diet (LC), and an LC with 15-minute post-meal walks (LC+Ex). The best results were observed for the LC+Ex diet (a significant reduction in glucose concentration was observed), and the worst results were presented for the GL diet. Moreover, a significant reduction in circulating proinsulin was observed for the LC and LC+Ex diets. Additionally, it was shown that the LC diet improved 4-day glycemic control and fasting proinsulin levels, compared to the GL diet. An additional 15-minute post-meal walk was beneficial with the LC diet [85].

### 6.3. The Effect of the Ketogenic Diet in the Therapy of Diabetes Type 2—Additional Studies

Two systematic reviews were not included in the PRISMA analysis due to their not being identified as a meta-analysis or systematic review by the PubMed filter [74] or not being listed in PubMed [75]. First, one systematic review from 2022 analyzed the influence of the ketogenic diet on insulin sensitivity in T2DM. It showed an improvement in glycemic control and the beneficial influence of the ketogenic diet on insulin sensitivity in type 2 diabetes [74]. The second article investigated the influence of the ketogenic diet on patients with metabolic disorders. Considering the parameters connected with diabetes, they reported an improvement in the concentrations of glycated hemoglobin, glucose, and insulin, and an improvement in HOMA-IR. Moreover, improvements in cholesterol, ALT, and AST concentrations were reported. [75] In 2022, an extensive meta-analysis was conducted, including 50 studies that involved 4291 patients, concerning the effect of a low-carbohydrate diet, in relation to the number of carbohydrates, in patients with type 2 diabetes. It should be stressed that the meta-analysis did not strictly concern the ketogenic diet. For that reason, the importance of that publication in the context of the ketogenic diet is not as reliable as that of the earlier-described meta-analyses of ketogenic diets. Taking into account the widely presented data in that meta-analysis, it is worth mentioning its results as a complement to the subject discussed herein. It was shown that over a period of six months, together with a reduction in the share of energy from carbohydrates from 55–65% to 10%, a linear reduction occurred in the glycated hemoglobin values (by 0.20 HBA1c %, on average), fasting glycemia (by 0.34 mmol/L, on average), body mass (by 1.44 kg, on average), triglyceride concentration (by 0.12 mmol, on average) and systolic blood pressure (by 1.79 mmHg, on average). From the 12-month perspective (from the 6th to the 12th month), the linear tendency was still observed in the glycated hemoglobin values (a reduction by 0.11 HbA1c %, on average) and in triglyceride concentration (reduction by 0.12 mmol, on average). A “U”-shaped effect after 12 months was noted in the case of body mass (the highest reduction being from a limitation of the carbohydrate energy share to 35%) and after 6 months in the case of total cholesterol and LDL fraction levels (the highest reduction was seen at a carbohydrate amount corresponding to 40% of the energy share) [86].

A one-year study of type 2 diabetes patients showed that owing to the ketogenic diet, fasting insulin values decreased by 43%, on average. Most of that effect had been noted already at the initial stage of 70 days [87]. Such benefits are not surprising when taking into account the character of the ketogenic diet. Not infrequently, its significant effect was demonstrated on the glycemic parameters, including the HOMA-IR (homeostasis model assessment of insulin resistance) index, which was established based on fasting glucose and insulin concentrations. In one study, it was already clear after 12 weeks that the index value was reduced from 3.73 to 1.4, on average [17]. The benefits resulting from diets, even with a moderate carbohydrate content (26–45% of the energy share from carbohydrates), low-carbohydrate content (11–25% of the energy share from carbohydrates), and very-low-carbohydrate content (<10% of the energy share from carbohydrates), have been shown in a significant number of research studies. Their effects in reducing the concentrations of glycated hemoglobin and triglycerides, blood pressure, body mass, and increasing HDL cholesterol concentrations have been shown [88,89,90,91,92,93]. Considering the presented data, it cannot be said that there is no evidence for the ketogenic diet’s effectiveness in type 2 diabetes. It is worth clearly stating that not all these effects were observed from the ketogenic diet. Some of them were related to the total amount of carbohydrates and not to the condition of ketosis. Although the number of significant clinical studies is not high, it is sufficient to conclude that the ketogenic diet can be extremely helpful. From the perspective of the currently available data, the potential benefits cannot be ignored. The topic definitely deserves attention and further development of the research in this field.

## 7. The Ketogenic Diet and Standard Recommended Diabetes Diets

**Figure 3:**
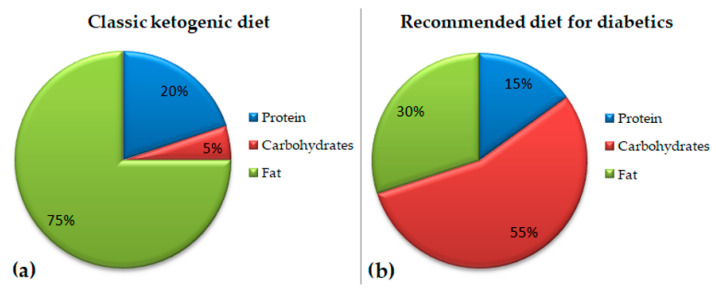
Comparison of an exemplary macronutrient ratio in the classic ketogenic diet (**a**) with that in an exemplary diabetic diet (**b**).

Although no ideal percentage proportions have been established for the calorie share from carbohydrates, fats, and protein to prevent diabetes, the recommended standard diets in patients with existing diabetes conditions are mainly based on the energy supplied by carbohydrates. The suggested amount is a 45–65% share of the energy supply. The recommended amount of fats is from 25% to 40% of the energy share, while that of protein is 15–20%. The maximal amount of cholesterol is 300 mg daily (in the case of dyslipidemia, it is <200 mg) [29,94]. Thus, the recommendations remain in contradiction to the principles of the ketogenic diet (the differences are illustrated in Figure 3). On the one hand, the observed common, although decreasing, reluctance toward employing the ketogenic nutritional model among some doctors and dieticians is not surprising. On the other hand, ignoring the current scientific evidence and potential benefits of such a diet and sticking to the old paradigm may be surprising. Moreover, when using that nutrition model, it is easier to control the glycemia level since the postprandial glucose and insulin levels are usually similar to the fasting ones, thus undergoing almost no changes [95]. Comparing studies on the ketogenic diet with the recommended standard diabetes diet model, it is clear that the ketogenic diet can produce even better effects, as has been described in the earlier parts of the current paper. In view of the literature, there is a chance that, over the years, the current dietary diabetic recommendations will possibly be modified.

## 8. The Ketogenic Diet in Practice

The implementation of the ketogenic diet should always be performed under the supervision of a dietician and medical doctor. Additionally, considering the high-fat and low-carbohydrate character of this diet, there is a limited base of usable products. The diet should be prepared with the elimination of starchy products (e.g., rice, groats, bread, and potatoes) and products with high amounts of simple sugars (e.g., sweets and fruits). Small amounts of fruits with low amounts of simple sugars (e.g., strawberries, raspberries, and blueberries) are allowed. Green leafy vegetables (e.g., arugula, spinach, kale, corn salad, and salad) are of high importance. The majority of vegetables, except scratch vegetables, can be put into the everyday menu. Meat, fish, eggs, and dairy products (to a lesser extent) are the source of protein, while avocado, olives, nuts, olive oil, cream, or butter will be good sources of fat. A proper selection of products rich in fatty acids (e.g., Omega-3) will provide an anti-inflammatory effect. Additionally, the level of hydration is significant. Special attention should be given to the number of meals planned for a diabetic patient, due to glucose fluctuations. The source of fluid should be water, but it can also include tea or coffee.

Due to the possible occurrence of the symptoms of ”keto-flu”, which arise as a result of increased water excretion, in the initial stage of the diet, it is beneficial to pay special attention to hydration and electrolyte (magnesium, sodium, and potassium) supplementation. Moreover, patients should regularly measure the glucose concentration in their blood and the ketone bodies in serum. In the initial stages of a ketogenic diet, diabetic patients should limit their physical activity to prevent potential hypoglycemic episodes. Patients taking medications and insulin should discuss their diet with their medical specialist, with further pharmacological management. It is possible to reduce the doses of drugs or to discontinue them altogether (e.g., insulin). It is worth mentioning that coming back to a high-carbohydrate diet may result in an increase in glycemia. Coming back to a high-carbohydrate diet should be gradual and should always be performed under the supervision of an experienced dietician and medical doctor.

## 9. Conclusions

Considering all the above-mentioned information concerning the effect of a ketogenic diet on the prophylaxis and treatment of diabetes, it can definitely be said that this is a promising direction for future research into the results derived from the low-carbohydrate character of a ketogenic diet, which induces a condition of ketosis in the body. The results of the current study can inspire optimism since the available data offer evidence of the favorable effect of a ketogenic diet on the prophylaxis and treatment of T2DM disease.

The perspective of drug-dose reduction or its complete withdrawal only renders the topic more interesting and calls for further exploration. Although carbohydrate reduction in the diet is beneficial on its own, it seems that the attainment of a ketosis state is necessary to obtain its therapeutic effects. This observation applies to both types of diabetes; however, the majority of data refers to T2DM.

Based on the current literature, the use of a ketogenic diet in T2DM treatment has its justifications.

The studies mentioned herein suggest an advantage of ketogenic diets over the standard diets recommended for patients with type 2 diabetes. The multifaceted benefits may outweigh those resulting from the standard dietary recommendations for diabetes patients. The observed beneficial effects of a ketogenic diet in T2DM are the reduction and stabilization of glucose and insulin concentrations in serum, the reduction of glycated hemoglobin concentration, and the reduction of the HOMAR-IR indicator, insulin resistance, and body mass. Additionally, the ketogenic diet shows anti-inflammatory effects and changes to the lipid parameters. It is worth mentioning that this effect is more visible for a ketogenic diet when combined with a caloric deficit. The available literature shows that the fears of potential adverse effects of using a ketogenic diet in T2DM patients are, in practice, disproportionate to the number of cases observed. With the correct monitoring of the patient’s condition, a skillfully adjusted diet poses no significant risk.

Based on the available data, it can be concluded that it may be reasonable to apply the ketogenic diet in type 1 diabetes (T1D). Although the literature evidence is limited, the beneficial effect of a ketogenic diet in T1D may arise as a consequence of its anti-inflammatory capabilities, glycemic stabilization, and potential effects on the pancreas.

According to the published studies, the occurrence of diabetic ketoacidosis or episodes of severe hypoglycemia is not frequent, although it should be taken into consideration, and the patient should be under medical supervision. It should be made clear, however, that further studies in this field are definitely needed. Although the available data bode well for the future, it seems that it is still too early to “give the green light” to the use of the ketogenic diet; this is caused by, e.g., the unavailability of long follow-up studies or a lack of final results.

In conclusion, the ketogenic diet may be advantageous in T2DM patients. In the case of T1D patients, the observed results are beneficial, but available data are limited and it is difficult to make a final assessment. The topic definitely deserves continued research and clinical follow-up. The results are promising enough to contribute not only to the development of science but also to a potential change in the recommendations for diabetes patients. Thus, it could improve the quality of life of many millions of individuals worldwide.

## Figures and Tables

**Table 1:** Effect of ketogenic diets as prescribed for children with type 1 diabetes.

Gender and Age	Disease	Dietary Intervention	Benefits	Adverse Effects	References
Boy, 4 years	Type 1 diabetes and myoclonic-astatic epilepsy	Ketogenic diet	1. Acceptable control of epileptic seizures 2. Improvement of cognitive functions 3. Maintenance of the target glycemia values	No severe episodes of hypoglycemia or ketoacidosis were observed	[56]
Girl, 14 years	Type 1 diabetes	Ketogenic diet	1. Significant improvement of subjective sensations 2. Significant improvement in glycemia	Not observed	[57]
Girl, 3.5 years	Type 1 diabetes, right hemiparesis, epilepsy	Ketogenic diet	1. Absence of epileptic seizures 2. Improvement of development, motor functions, and activity 3. Proper glycemic control and improvement in the HbA1c value	1 episode of ketoacidosis, apart from which no adverse effects were observed	[58]
A 9-year-old child (no information on gender)	Type 1 diabetes	Paleolithic ketogenic diet	1. Improvement of insulin and glucose levels 2. Discontinuation of insulin treatment	Not observed	[59]
Girl, 2 years	Type 1 diabetes and epilepsy	Ketogenic diet	1. No episodes of epileptic seizures 2. Improvement (reduction) of glycated hemoglobin level 3. No new episodes of diabetic ketoacidosis	Mild hypoglycemia episodes	[60]
Girl, 4 years	Pyruvate dehydrogenase deficiency, diabetic ketoacidosis, static encephalopathy, convulsive disorders	Ketogenic diet	1. Proper glycemic control 2. Improvement of activity level 3. Significant developmental achievement 4. Compensation of linear growth from < 5th to the 50th percentile	No major adverse effects were observed	[9]

**Table 2:** Effect of a ketogenic diet in adults with type 1 diabetes.

Gender and Age	Disease	Dietary Intervention	Benefits	Adverse Effects	References
Group of 17 women and 7 men. Mean age 51 ± 10 years	Type 1 diabetes	Low-carbohydrate diet (70–90 g carbohydrates)	1. Significant reduction in hypoglycemia episodes and glycated hemoglobin concentration (from 7.5% to 6.4%), 2. Significant reduction in the postprandial requirement for insulin (from 21.1 IU daily to 12.4 IU daily) 3. Reduction of triglyceride concentration by 16% on average	Diabetic gastroparesis was observed in 6 individuals	[62]
Group of 7 men and 4 women. Mean age 36.1 ± 6.8 years	Type 1 diabetes	Ketogenic diet	1. Normal HbA1c concentration (The mean HbA1c levels were 35 ± 4 mmol/mol (5.3 ± 0.4%)) 2. Slight changes in glycemia values (little daily glycemic variability (1.5 ± 0.7 mmol/L)	Hypoglycemia episodes and dyslipidemia were observed	[63]
Group of 4 men and 1 woman. Mean age 44.5 ± 10.4 years (in the normal carbohydrate amount group, there were an additional 3 men and 2 women, mean age 44.8 ± 8.3 years)	Type 1 diabetes	Target: ketogenic diet, 50–75 g carbohydrates as a result: low-carbohydrate diet, up to 100 g of carbohydrates	1. Improved glycemic control (significant reductions in HbA1c (63 to 55 mmol/mol)) 2. Reduction of insulin doses (significant reductions in daily insulin use (64.4 to 44.2 units/day)) 3. Body mass reduction (83.2 to 78.0 kg)	One participant reported a higher irritability and another one a greater number of minor diseases	[64]
Group of 316 women and men. Mean age 27 ± 19	Type 1 diabetes	Carbohydrates of 36 g on average, i.e., a ketogenic diet	1. Improved glycemic control (mean HbA1c was 5.67% ± 0.66%) 2. Lower requirement for insulin	A group of 7 individuals in a year, who reported a hospitalization associated with diabetes (including ketoacidosis and hypoglycemia); in the remaining majority of cases, there were no adverse effects	[65]
Man. 37 years	Type 1 diabetes	Ketogenic diet	1. Maintenance of glycemic stability (average interstitial glucose 6.1 mmol/L) and 80.4% of the time spent within a range of 3.9–10 mmol/L. Interstitial glucose was < 3 mmol/L for 2.1% of this time 2. No problems with riding 4011 km on a bicycle over 20 days	1 episode of major hypoglycemia	[66]
Woman. 22 years	Undiagnosed type 1 diabetes	Ketogenic diet	None	Development of diabetic ketoacidosis in 4 days	[67]

**Table 3:** Summary of the meta-analyses and systematic reviews concerning the effect of a ketogenic diet on patients with type 2 diabetes.

Year and Type of Publication	Number of Studies Considered	Diet Type	Publication Aim	Results	References
2020 Meta-analysis	14 RCT	Ketogenic diet	Assessment of the effectiveness of a ketogenic diet in metabolic compensation in patients who are overweight/obese, with and without type 2 diabetes, compared with a low-fat diet	Advantages of a ketogenic diet over a low-fat diet in the control of glycemia (lower HbA1c levels (SMD −0.62), body mass (SMD −0.46) and lipid profile (reduction in triglyceride concentration (mean -0.45, increase in HDL (SMD 0.31) concentration)	[68]
2020 Systematic review and meta-analysis	13	Ketogenic diet	Assessment of the effect of a ketogenic diet on the control of glycemia, insulin resistance, and lipid metabolism in type 2 diabetes patients	Reduction in the concentrations of glucose (by 1.29 mmol/L on average), glycated hemoglobin (by 1.07% HbA1c on average), total cholesterol (by 0.33 mmol/L on average, LDL (by 0.05 mmol/L on average), and the reduction of body mass (by 8.66 kg on average), waist circumference (by 9.17 cm on average), and BMI (by 3.13 kg/m^2^ on average) HDL concentration increased (by 0.14 mmol/L, on average)	[69]
2022 Meta-analysis	8 RCT	Ketogenic diet	Studying the role of the ketogenic diet in controlling body mass and glycemia in patients with type 2 diabetes who are overweight	Reduction in body mass (by 5.63 kg on average), waist circumference (by 2.32 cm on average), glycated hemoglobin concentration (by 0.38% HbA1c on average), triglycerides (by 0.36 mmol/L on average) and an increase in HDL cholesterol concentration (by 0.28 mmol/L, on average)	[70]
2022 Meta-analysis	10 RCT	Ketogenic diet	Assessment of the effect of a ketogenic diet on lipid metabolism in patients with type 2 diabetes (compared with the effects of the standard diets)	Advantage of the ketogenic diet in reducing triglyceride concentration, particularly in the 3rd month (compared with the control group, with TG reduction in the 3rd, 6th, and 12th months of treatment, by 0.49 mmol/L, −0.82 mmol/L, and −0.18 mmol/L, on average) No significant differences in total cholesterol, LDL, and HDL concentrations	[76]
2022 Systematic review and meta-analysis	8 RCT	Ketogenic diet	Estimation of the effect of a ketogenic diet in type 2 diabetes patients and individuals with pre-diabetic conditions (compared with the diets with a higher content of carbohydrates than in a ketogenic diet)	In individuals on a ketogenic diet, compared with the control group, a reduction of triglyceride concentration by 0.28 mmol/L and an increase in HDL cholesterol level by 0.04 mmol/L. In total, 4 studies demonstrated changes in HbA1c concentration after 12 months, with the estimation of persistent effects (VLC/KD minus control group) at 0.01% level (−0.22 to 0.25). In addition, 2 studies demonstrated a change in HbA1c from the initial value: −0.65% (−0.99; −0.31)	[71]
2022 Meta-analysis	8RCT	Ketogenic diet	Comparison of a ketogenic diet vs. standard diet recommended in patients with type 2 diabetes in the context of parameter changes, i.e.: glycemia, body mass, lipid profile, drug taking, and the discontinuation of drug taking	Compared with the standard recommended diets, in patients on a ketogenic diet, a reduction in HbA1c after 3 and 6 months (by 6.7 mmol/L and 6.3 mmol/L, on average, respectively) and a body mass reduction after 3 and 6 months (by 2.91 kg and 2.84 kg on average, respectively) were observed. In a 12-month period, an advantage of a ketogenic diet over the control diets was seen in respect of triglyceride concentration reduction and a reduction in the requirements for drugs, as well as an increase in HDL concentration	[72]
2022 Systematic review and meta-analysis	15	Ketogenic diet and low-carbohydrate diet	Assessment of the effectiveness of a ketogenic diet (and low-carbohydrate diet) in controlling glycemia and body mass in patients with type 2 diabetes	Patients using a ketogenic diet, compared with control diets, reduced their glycated hemoglobin values by 1.45% HbA1c, on average, and their body mass by 2.67 kg, on average	[73]
2021 Systematic review	14	Ketogenic diet	Assessment of the pleiotropic effect of a ketogenic diet on glycemic control, drug changes, and body mass loss in patients with type 2 diabetes	Improvement of glycated hemoglobin concentrations in patients with type 2 diabetes within three weeks and the persistence of the effect for at least one year. Effectiveness in the long-term maintenance of a reduced body mass	[39]

**Table 4:** Summary of the randomized controlled trials (RCT), listing the effect of a ketogenic diet on patients with type 2 diabetes.

Year and Type of Publication	Number of Patients and Duration	Diet Type	Publication Aim	Main Outcome(for Ketogenic Diet)	Percentage of Drop-Outs	References
2017, RCT	25 (12 in the intervention, 13 control). 32 weeks	Ketogenic diet ad libitum vs. a diet program based on the American Diabetes Association’s “Create Your Plate” website	Comparison of the online intervention of a ketogenic diet ad libitum vs. an online diet program based on the American Diabetes Association’s “Create Your Plate” website on glycemic control and other health outcomes among overweight individuals with type 2 diabetes	1. Greater HbA1c decrease (−0.8% vs. −0.6%), 2. Lowering HbA1c to less than 6.5% (55% of participants vs. 0% of participants), 3. Greater weight loss (−12.7 kg vs. −3.0 kg), 4. A greater percentage of participants lost at least 5% of their body weight (90% of participants vs. 29% of participants) 5. Greater reduction in triglyceride levels (−60,1mg/dl vs. −28,9mg/dl) 6. Lower numbers of dropouts (8% of participants vs. 46% of participants)	Intervention group: 8% (1/12) and control group: 46% (6/13)	[78]
2016, RCT	89 (45 in the intervention, 44 control). 4 months	Low-calorie ketogenic diet (VLC/KD) vs. a standard low-calorie diet	Evaluating the short-term safety and tolerability of a very low-calorie-ketogenic diet (VLCKD) (< 50 g of carbohydrate daily) in an interventional weight loss program including lifestyle and behavioral modification support (Diaprokal method) in subjects with T2DM.	1. Greater body mass reduction (−14.7 kg vs. −5.05 kg) 2. Greater percentage of participants lost more than 5% and 10% of their body weight (97.6% vs. 50% and 85.4% vs. 16.7%, respectively) 3. Greater reduction in waist circumference (−12 cm vs. −5.4 cm) 4. Greater reduction in HbA1c levels (−0.9% vs. −0.4%) 5. Greater reduction in oral diabetes medication (from 73.3% to 50% of participants vs. from 86.4% to 83.3% of participants)	VLCKD: 11.1% (5/45), LC diet: 18.2% (8/44)	[79]
2022, RCT	60 (30 intervention, 30 control). 12 weeks	Ketogenic diet vs. the routine diet for diabetes	Observation of a periodic ketogenic diet for its effect on overweight or obese patients newly diagnosed with T2DM, with a comparison with the routine diet for diabetes	1. Greater reduction in HbA1c levels (−0.92% vs.–0.27%) 2. Greater reduction in fasting glucose concentration (−1.39 mmol/L vs. −0.56 mmol/L) 3. Greater reduction in fasting insulin concentration (−48.23 pmol/L vs. −3.7 pmol/L) 4. Greater body mass reduction (−8.06 kg vs. −0.61 kg) 5. Greater reduction in waist circumference (−9.29 cm vs. −0.77 cm) 6. Increase HDL concentration (+0.13 mmol/L vs. + 0.03 mmol/L) and decrease of LDL concentration (−0.41 mmol/L vs. −0.18 mmol/L)	Ketogenic diet group: 20% (6/30), Control group: 3.3% (1/30)	[80]
2022, RCT	40 (20 + 20) (33 after drop-outs (16 on the ketogenic diet vs. 17 on the Mediterranean diet)). 12 weeks	Ketogenic diet vs. a low-carb Mediterranean diet	Comparison of 2 low-carbohydrate diets with 3 key similarities (incorporating nonstarchy vegetables and avoiding added sugars and refined grains) and 3 key differences (incorporating, compared with avoiding, legumes, fruits, and whole, intact grains) for their effects on glucose control and cardiometabolic risk factors in individuals with pre-diabetes and T2DM	1. Decrease in triglyceride concentration (−16% vs. −5%) 2. Increase in HDL concentration (+11% vs. +7%) 3. Greater reduction in body mass (−8% vs. −7%) 4. Increase in LDL concentration (+10% vs. −5%)	17.5% (7/40)	[81]
2021, RCT	40 (20 + 20) 24 weeks (12 + 12)	Ketogenic diet vs. a low-carb Mediterranean diet	Detailed examination and comparison of the adherence to the two study diets (well-formulated ketogenic diet (WFKD) and Mediterranean Plus (Med-Plus) under two conditions: all food being provided (delivered) and all food being obtained by individual participants (self-provided)	1. Higher adherence to the WFKD diet in the food delivery phase (7.6 vs. 7.3) and self-provided food (5.7 vs. 5.4) on a 10-point scale. 2. After study completion, a clear relationship with diet preference was observed—participants preferred the diet that they were assigned first	12.5% (5/40)	[84]
2014, RCT	34 (16 ketogenic diet + 18 standard ADA diet). 3 months	Ketogenic diet vs. a diet consistent with guidelines from the American Diabetes Association	Comparison of the effects of each diet on glycemic control, medication use, and weight loss among overweight or obese individuals with type 2 diabetes mellitus or prediabetes + testing the feasibility of research design for conducting a larger scale	1. Decrease in HbA1c levels (−0.6% vs. 0%) 2. Higher percentage of participants discontinued one or more diabetes medications (44% vs. 11%) 3. Higher percentage of participants discontinued sulfonylureas (31% vs. 5%) 4. Greater body mass reduction (−5.5 kg vs. −2.6 kg)	LCK group: 6.25% (1/16), MCCR group: 5.55% (1/18)	[82]
2017, RCT	34 (16 ketogenic diet vs. 18 standard diet). 12 months	Ketogenic diet vs. a low fat, moderate carbohydrate, calorie-restricted diet	Comparison of the effects of each diet on glycemic control, medication use, and weight loss among overweight or obese individuals with type 2 diabetes mellitus or pre-diabetes	1. Greater reduction in HbA1c levels (−0.5% vs. −0.2%) 2. Greater body mass reduction (−7.9 kg vs. −1.7 kg) 3. Discontinued metformin medication (30% of participants vs. 0% of participants) 4. Discontinued sulfonylureas or dipeptidyl peptidase-4 inhibitor medications (60% of participants vs. 0% of participants)	LCK group: 12.5% (2/16), MCCR group: 16.7% (3/18)	[83]

## Data Availability

Not applicable.
